# Planktonic and Biofilm-Derived Pseudomonas aeruginosa Outer Membrane Vesicles Facilitate Horizontal Gene Transfer of Plasmid DNA

**DOI:** 10.1128/spectrum.05179-22

**Published:** 2023-03-22

**Authors:** Ella L. Johnston, Lauren Zavan, Natalie J. Bitto, Steve Petrovski, Andrew F. Hill, Maria Kaparakis-Liaskos

**Affiliations:** a Department of Microbiology, Anatomy, Physiology and Pharmacology, School of Agriculture, Biomedicine and Environment, La Trobe University, Melbourne, Victoria, Australia; b Research Centre for Extracellular Vesicles, La Trobe University, Melbourne, Victoria, Australia; c Department of Biochemistry and Chemistry, School of Agriculture, Biomedicine and Environment, La Trobe University, Melbourne, Victoria, Australia; d Institute for Health and Sport, Victoria University, Melbourne, Victoria, Australia; LMU Munich

**Keywords:** bacterial membrane vesicles, OMVs, horizontal gene transfer, bacterial growth conditions, plasmid

## Abstract

Outer membrane vesicles (OMVs) produced by Gram-negative bacteria package various cargo, including DNA that can be transferred to other bacteria or to host cells. OMV-associated DNA has been implicated in mediating horizontal gene transfer (HGT) between bacteria, which includes the dissemination of antibiotic resistance genes within and between bacterial species. Despite the known ability of OMVs to mediate HGT, the mechanisms of DNA packaging into OMVs remain poorly characterized, as does the effect of bacterial growth conditions on the DNA cargo composition of OMVs and their subsequent abilities to mediate HGT. In this study, we examined the DNA content of OMVs produced by the opportunistic pathogen Pseudomonas aeruginosa grown in either planktonic or biofilm conditions. Analysis of planktonic growth-derived OMVs revealed their ability to package and protect plasmid DNA from DNase degradation and to transfer plasmid-encoded antibiotic resistance genes to recipient, antibiotic-sensitive P. aeruginosa bacteria at a greater efficiency than transformation with plasmid alone. Comparisons of planktonic and biofilm-derived P. aeruginosa OMVs demonstrated that biofilm-derived OMVs were smaller but were associated with more plasmid DNA than planktonic-derived OMVs. Additionally, biofilm-derived P. aeruginosa OMVs were more efficient in the transformation of competent P. aeruginosa bacteria, compared to transformations with an equivalent number of planktonic-derived OMVs. The findings of this study highlight the importance of bacterial growth conditions for the packaging of DNA within P. aeruginosa OMVs and their ability to facilitate HGT, thus contributing to the spread of antibiotic resistance genes between P. aeruginosa bacteria.

**IMPORTANCE** Bacterial membrane vesicles (BMVs) mediate interbacterial communication, and their ability to package DNA specifically contributes to biofilm formation, antibiotic resistance, and HGT between bacteria. However, the ability of P. aeruginosa OMVs to mediate HGT has not yet been demonstrated. Here, we reveal that P. aeruginosa planktonic and biofilm-derived OMVs can deliver plasmid-encoded antibiotic resistance to recipient P. aeruginosa. Additionally, we demonstrated that P. aeruginosa biofilm-derived OMVs were associated with more plasmid DNA compared to planktonic-derived OMVs and were more efficient in the transfer of plasmid DNA to recipient bacteria. Overall, this demonstrated the ability of P. aeruginosa OMVs to facilitate the dissemination of antibiotic resistance genes, thereby enabling the survival of susceptible bacteria during antibiotic treatment. Investigating the roles of biofilm-derived BMVs may contribute to furthering our understanding of the role of BMVs in HGT and the spread of antibiotic resistance in the environment.

## INTRODUCTION

Bacteria have long been recognized to have immense genetic plasticity, which contributes to their ability to survive and adapt in various environments (1–3). Horizontal gene transfer (HGT), the transfer of genetic information between bacteria, is vital for the adaptation and evolution of bacteria. The main mechanisms of HGT include conjugation, natural transformation, and transduction (1). Recently, a fourth mechanism of HGT was recognized, that which is mediated by bacterial membrane vesicles (BMVs) (4–12). BMVs are nanoparticles naturally produced by bacteria as part of their normal growth, and they are broadly classified as outer membrane vesicles (OMVs) and membrane vesicles (MVs) when released by Gram-negative and Gram-positive bacteria, respectively, of which there are multiple subtypes within these populations (13–16). BMVs have been shown to package various types of DNA, including plasmid, chromosomal, and bacteriophage DNA (6, 17, 18), and can protect DNA from degradation, thus contributing to the persistence of extracellular DNA in the environment and in mediating HGT (19, 20).

The ability of BMVs to mediate HGT has been demonstrated for a limited number of bacterial species, which includes pathogenic Escherichia coli, Porphyromonas gingivalis, Acinetobacter spp., and Ruminococcus albus (5–10). However, despite DNA being packaged within BMVs produced by several other bacterial species, including Streptococcus mutans, Pseudomonas aeruginosa, and *Prochlorococcus* spp., their ability to mediate HGT has not been shown (18, 19, 21), and numerous attempts to demonstrate HGT mediated by P. aeruginosa OMVs have been unsuccessful (21). Furthermore, bacteria can vary the quantity and composition of BMVs produced during various growth conditions and stages of bacterial growth (22), which includes increasing their DNA content when produced during biofilm growth conditions (23). Despite our growing understanding of the regulation of BMV composition when produced by bacteria during various growth conditions, it is unknown whether bacterial growth conditions affect the ability of BMVs to mediate HGT, and therefore the ability of P. aeruginosa to be transformed using BMVs produced during different conditions of bacterial growth remains unknown.

In this study, we examined the ability of BMVs produced during planktonic and biofilm growth conditions to facilitate HGT. To do this, BMVs derived from P. aeruginosa grown during either planktonic or biofilm conditions, which we refer to as OMVs throughout this study, were compared for their ability to package plasmid DNA encoding antimicrobial resistance and to protect DNA from DNase treatment. Furthermore, the ability of planktonic-derived OMVs and biofilm-derived OMVs to transfer plasmid-encoded antibiotic resistance genes to recipient competent P. aeruginosa was examined. We found that planktonic-derived OMVs contained plasmid DNA which was transferred to recipient P. aeruginosa more efficiently compared to transformation with plasmid DNA alone. We identified that biofilm-derived P. aeruginosa OMVs contained more plasmid DNA compared to planktonic-derived OMVs, which was predominantly on the surface of biofilm-derived OMVs. Moreover, we found that biofilm-derived OMVs were more efficient in mediating HGT of plasmid DNA encoding antibiotic resistance compared to planktonic-derived OMVs. Overall, these findings significantly advance our knowledge of the regulation of DNA packaged within P. aeruginosa OMVs and identify the impact of bacterial growth conditions on determining the composition of OMVs and their efficiency to mediate HGT and promote antimicrobial resistance.

## RESULTS

### Pseudomonas aeruginosa grown in planktonic conditions produce OMVs containing plasmid DNA.

**FIG 1:**
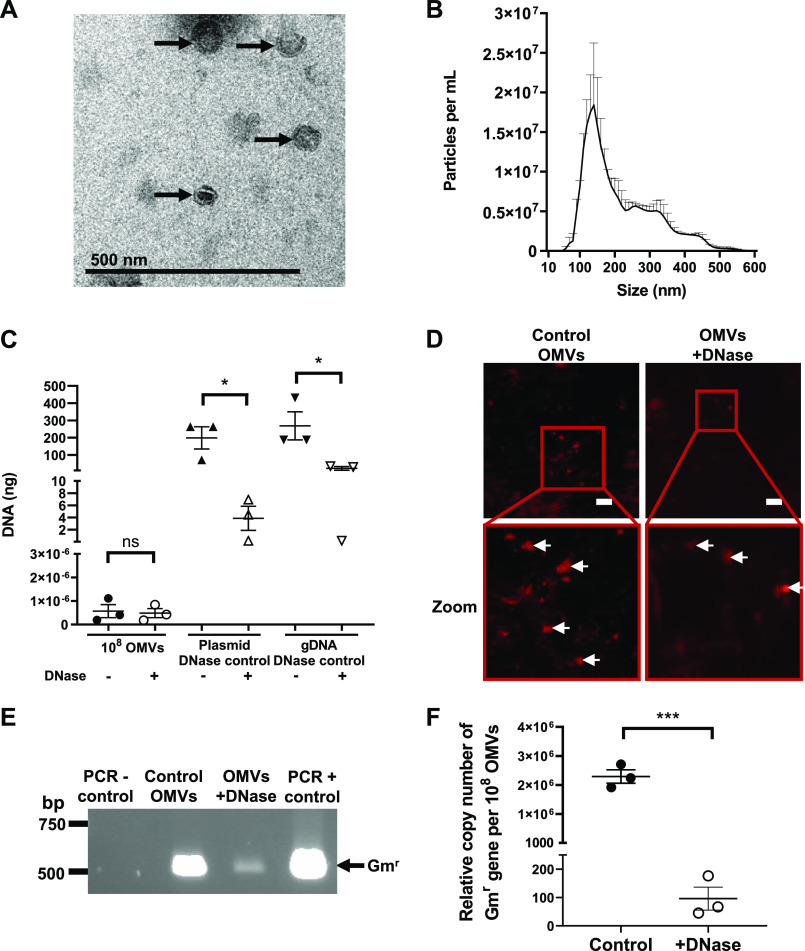
Planktonic-derived P. aeruginosa PAO9505 OMVs package and protect plasmid DNA. (A) Transmission electron micrograph of PAO9505 pBBR1MCS-5-containing OMVs produced during planktonic growth. Scale bar, 500 nm. Image is representative of *n* = 3 biological replicates. Arrows indicate OMVs. (B) The size and concentration of planktonic-derived PAO9505 OMVs containing pBBR1MCS-5 were determined by NanoSight NTA. Results shown are means + SEM of *n* = 3 biological replicates. (C) DNA (in nanograms) associated with 10^8^ control (−; filled shapes) or DNase-treated (+; open shapes) planktonic-derived PAO9505 OMVs (circles) harboring pBBR1MCS-5 was quantified using Qubit. As a control for DNase treatment, pBBR1MCS-5 plasmid DNA (plasmid DNase control; triangles) and P. aeruginosa gDNA (gDNA DNase control; downward triangles) were treated with DNase (+) or not treated, as controls (−), and DNA was quantified. Data are the means ± SEM of *n* = 3 biological replicates. ns, not significant; *, *P < *0.05 (Student's *t* test). (D) Control and DNase-treated P. aeruginosa PAO9505 OMVs were stained using the DNA stain SYTO-61 (red) and imaged using confocal microscopy. Red boxes show the area that is magnified in the image below (zoom). White arrows indicate SYTO-61-stained DNA. Scale bar, 10 μm. Images are representative of *n* = 3 biological replicates. (E) PCR of control and DNase-treated OMVs isolated from planktonic PAO9505 pBBR1MCS-5 cultures using primers specific for the gentamicin resistance cassette (Gm^r^). H_2_O was used as a negative control (PCR − control) and pBBR1MCS-5 was used as a positive control (PCR + control) for the PCR. Arrow indicates the gentamicin cassette amplicon (Gm^r^; 500 bp). Image is representative of *n* = 3 biological replicates. (F) The relative copy number of the Gm^r^ gene from pBBR1MCS-5 associated with 10^8^ control or DNase-treated OMVs isolated from planktonic cultures of PAO9505 harboring pBBR1MCS-5 was determined by qPCR and used as an indicator of plasmid copy number. Data are means ± SEM of *n* = 3 biological replicates. ***, *P = *0.001 (Student's *t* test).

P. aeruginosa has previously been demonstrated to release OMVs that package and protect DNA, including plasmid DNA (21). However, despite numerous attempts, the ability of P. aeruginosa OMVs to mediate HGT has not been demonstrated (21). To examine the ability of P. aeruginosa OMVs to mediate HGT, OMVs were isolated from P. aeruginosa PAO9505 that was transformed with the low-copy and broad-host-range plasmid pBBR1MCS-5, encoding gentamicin resistance (24). OMVs derived from planktonic cultures of P. aeruginosa PAO9505 harboring pBBR1MCS-5 were examined using transmission electron microscopy (TEM) and NanoSight nanoparticle tracking analysis (NTA) to determine their morphology, purity, size, and quantity (Fig. 1A and B). Planktonic-derived P. aeruginosa PAO9505 OMVs ranged between 50 and 500 nm in size with the predominant population of OMVs being approximately 100 to 200 nm in size, as determined by NTA (Fig. 1B).

We next examined the quantity of DNA packaged into planktonic-derived P. aeruginosa OMVs. To remove extravesicular DNA, OMVs were treated with DNase and were compared to non-DNase-treated control OMVs. As controls for the effectiveness of the DNase treatment, pBBR1MCS-5 plasmid DNA and P. aeruginosa genomic DNA (gDNA) were also treated with DNase (Fig. 1C). DNase treatment did not significantly alter the amount of DNA associated with DNase-treated and control OMVs (Fig. 1C), suggesting that a large proportion of DNA was packaged within planktonic derived P. aeruginosa OMVs, which has also been reported previously (20). To further examine the location of DNA associated with planktonic-derived OMVs, DNase-treated or control OMVs were stained using the membrane-permeable fluorescent DNA stain SYTO-61 and were visualized using confocal microscopy (Fig. 1D). Examination of SYTO-61-stained control and DNase-treated OMVs revealed that DNA remained within OMVs after DNase treatment, as well as being on the surface of control OMVs, suggesting that DNA was protected from degradation due to its packaging within planktonic-derived P. aeruginosa OMVs (Fig. 1D).

Having confirmed the presence of bacterial DNA on the surface and within P. aeruginosa OMVs, the ability of planktonic-derived OMVs to package and protect plasmid DNA encoding gentamicin resistance (Gm^r^) from degradation was further examined. The presence of the pBBR1MCS-5 plasmid associated with control P. aeruginosa OMVs and within the lumen of DNase-treated OMVs was confirmed by PCR using primers specific for the Gm^r^ cassette encoded by pBBR1MCS-5 (Fig. 1E). Finally, quantification of the relative copy number of Gm^r^ genes carried by pBBR1MCS-5, used as a measure of plasmid copy number, associated on the surface and within 10^8^ control and DNase-treated OMVs was determined by quantitative PCR (qPCR). This revealed that P. aeruginosa control OMVs were associated with significantly more copies of the Gm^r^ gene from pBBR1MCS-5 compared to DNase-treated OMVs, and therefore that DNase-treated OMVs were able to protect plasmid from DNase degradation when contained within the OMV lumen (Fig. 1F) (*P < *0.001).

### Planktonic-derived P. aeruginosa OMVs transfer extravesicular plasmid DNA to recipient bacteria more efficiently than when transforming with plasmid alone.

**FIG 2:**
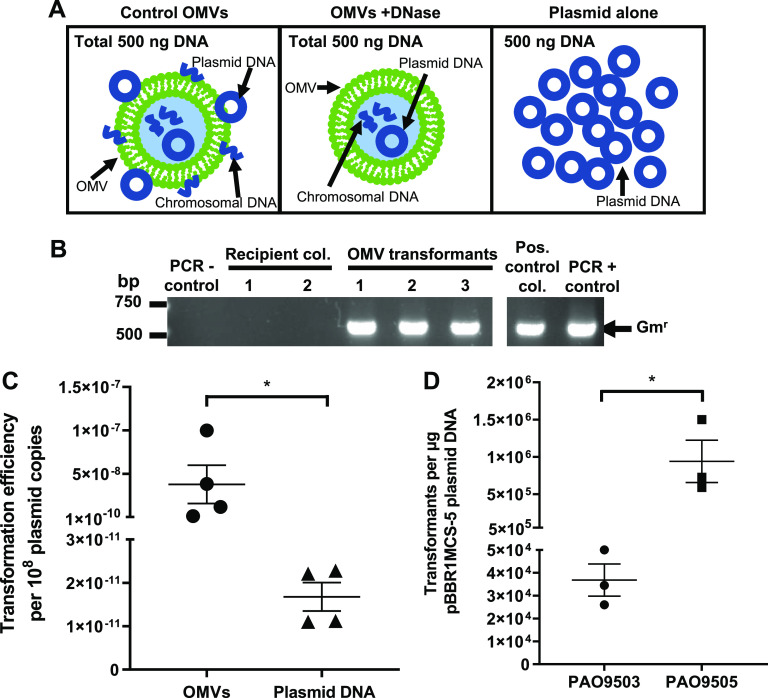
Planktonic-derived P. aeruginosa PAO9505 OMVs transfer plasmid DNA to recipient bacteria more efficiently than transformation with plasmid DNA alone. (A) Schematic diagram illustrating transformation of bacteria with 500 ng of total DNA associated with either non-treated control OMVs, which included extravesicular chromosomal and plasmid DNA (control OMVs), DNase-treated OMVs, which contained plasmid and chromosomal DNA (OMVs +DNase), or 500 ng of plasmid alone (plasmid alone). (B) The presence of pBBR1MCS-5 within PAO9503 transformants resulting from transformation using either control PAO9505 OMVs or pBBR1MCS-5 plasmid DNA alone as a positive control was confirmed by colony PCR using primers specific for the detection of the pBBR1MCS-5 gentamicin resistance cassette (Gm^r^). A positive plasmid control PAO9503 transformant (pos. control col.) and pBBR1MCS-5 DNA (PCR + control) were used as positive controls for the PCR. H_2_O (PCR − control) and PAO9503 recipient colonies (recipient col) were used as negative controls for the PCR. Arrow indicates the gentamicin cassette amplicon (Gm^r^; 500 bp). Data are representative of images from *n* = 4 biological replicates. (C) The transformation efficiency of control PAO9505 pBBR1MCS-5 OMVs (circles) and plasmid DNA alone (triangles) was calculated as the number of PAO9503 transformants per 10^8^ plasmid DNA copies. Data are means ± SEM of *n* = 4 biological replicates. *, *P < *0.05 (Mann-Whitney test). (D) The number of transformants resulting from transformation of P. aeruginosa PAO9503 or PAO9505 with 1 μg of pBBR1MCS-5. Data are means ± SEM of *n* = 3 biological replicates. *, *P < *0.05 (Student's *t* test).

Having shown that P. aeruginosa OMVs were associated with and contained plasmid DNA, we next examined the ability of P. aeruginosa OMVs to facilitate HGT. To do this, competent P. aeruginosa PAO9503 bacteria were incubated with DNase-treated or control P. aeruginosa PAO9505 OMVs harboring pBBR1MCS-5, each containing a total of 500 ng of DNA, and their ability to mediate HGT was examined (Fig. 2A). As a positive control for the transformation, 500 ng of plasmid DNA was added to recipient P. aeruginosa PAO9503, whereas the addition of Tris-EDTA (TE) buffer to P. aeruginosa PAO9503 served as a negative control.

P. aeruginosa PAO9503 was successfully transformed using control PAO9505 OMVs harboring a total of 500 ng of DNA located both on the surface and within OMVs. No transformants were obtained when P. aeruginosa PAO9503 was incubated with DNase-treated OMVs containing a total of 500 ng of DNA, suggesting that OMVs facilitate the transfer of plasmid DNA bound to their exterior surface to recipient P. aeruginosa bacteria. In comparison, transformants were successfully obtained when P. aeruginosa PAO9503 was transformed using 500 ng of pBBR1MCS-5 plasmid DNA, and no transformants resulted from incubation with TE buffer. Transformants were confirmed by performing colony PCR, resulting in amplification of the Gm^r^ cassette encoded by pBBR1MCS-5, which was absent from all negative controls (Fig. 2B). Collectively, these findings revealed that P. aeruginosa OMVs harboring plasmid DNA both on their surface and within their lumen could successfully transform P. aeruginosa PAO9503, whereas plasmid DNA contained within DNase-treated P. aeruginosa OMVs could not mediate HGT.

Next, we determined the efficiency of transformation mediated by control OMVs containing a total of 500 ng of DNA compared to transformation of P. aeruginosa with 500 ng of plasmid alone. To do this, the transformation efficiency was determined as the number of transformants resulting from transformation with 10^8^ plasmid copies either associated with OMVs or using purified plasmid (Fig. 2C). Control OMVs contained substantially less plasmid DNA, with approximately 1 × 10^6^ plasmid copies per 500 ng of total DNA being associated with approximately 5 × 10^7^ control OMVs, as calculated using qPCR, compared to 500 ng of plasmid DNA, corresponding to approximately 1 × 10^11^ plasmid copies. However, despite containing less plasmid DNA, P. aeruginosa OMVs had a significantly higher transformation efficiency compared to transformation with plasmid DNA (Fig. 2C) (*P < *0.05).

As transformation efficiencies differ between P. aeruginosa strains when transformed with plasmid alone (25, 26), we next examined whether there were strain differences in the number of transformants that resulted from transformation of PAO9503 and PAO9505 with pBBR1MCS-5 plasmid DNA alone. We found that transformation of PAO9505 with pBBR1MCS-5 resulted in significantly more transformants compared to PAO9503 (Fig. 2D) (*P < *0.05); therefore, all subsequent studies examining the characterization and transformation efficiencies of P. aeruginosa OMVs used PAO9505 as the recipient strain to maximize our ability to observe OMV-mediated HGT in the laboratory setting.

### Biofilm-derived P. aeruginosa OMVs are smaller than planktonic-derived P. aeruginosa OMVs.

Recent studies have shown that OMVs isolated from biofilm cultures contain significantly more DNA than OMVs isolated from planktonic cultures and that they also differ in their size and packaging of content (23, 27, 28). However, differences in the ability of OMVs derived during biofilm or planktonic growth conditions to mediate HGT have not been examined. Therefore, we isolated OMVs from planktonic or biofilm cultures of P. aeruginosa PAO9503 harboring pBBR1MCS-5 to compare their DNA quantities, size, and abilities to package plasmid DNA and to mediate HGT.

**FIG 3:**
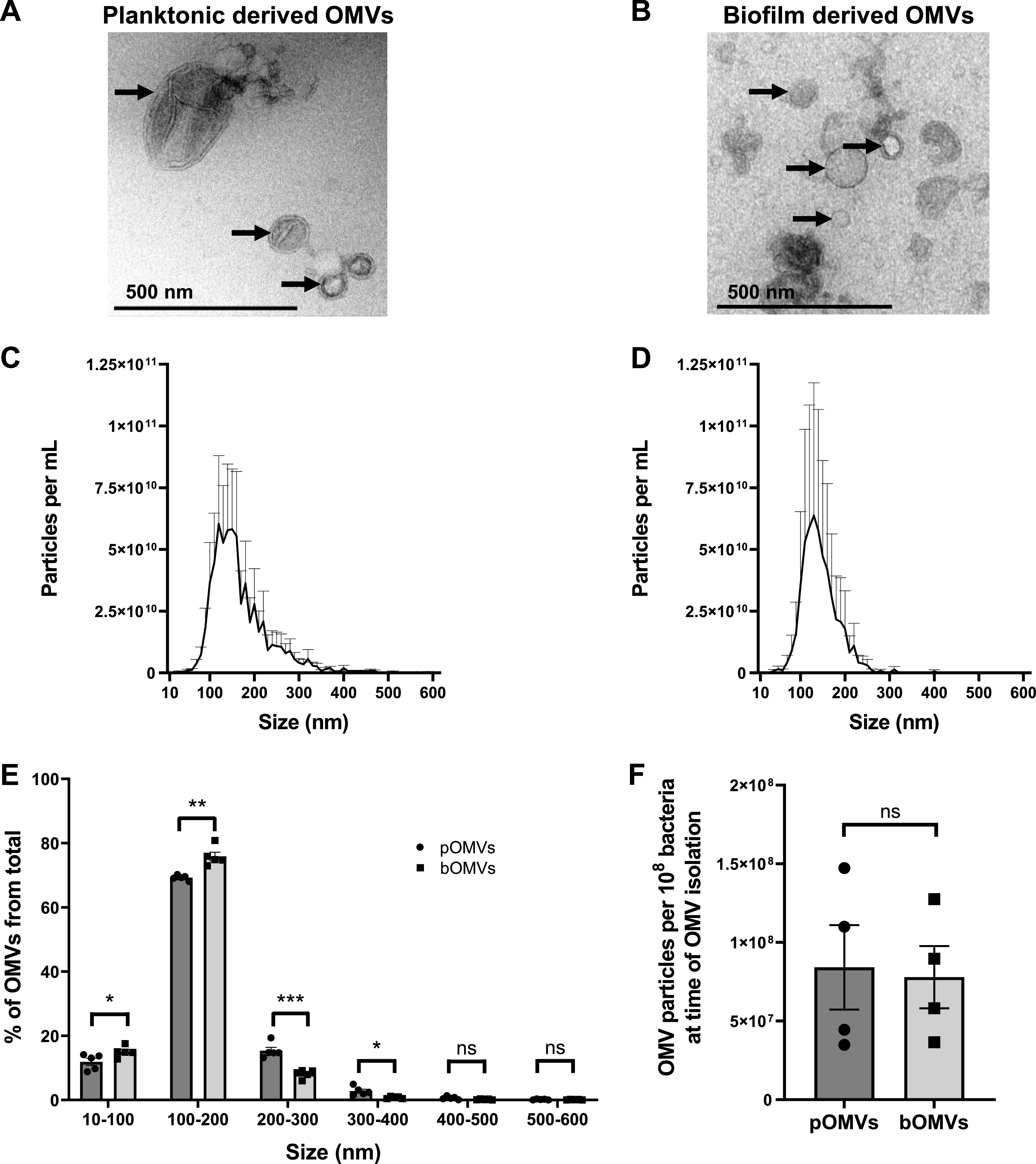
P. aeruginosa PAO9503 OMVs produced during biofilm growth are smaller than planktonic-derived P. aeruginosa PAO9503 OMVs. (A and B) Transmission electron micrographs of PAO9503 pBBR1MCS-5 planktonic derived OMVs (A) and PAO9503 pBBR1MCS-5 biofilm derived OMVs (B). Scale bar, 500 nm. Images are representative of *n* = 3 biological replicates. Arrows indicate OMVs. (C and D) ZetaView NTA of OMVs isolated from PAO9503 pBBR1MCS-5 planktonic (C) or biofilm (D) cultures. Data shown are means + SEM of *n* = 5 biological replicates. (E) The size range of P. aeruginosa PAO9503 planktonic derived OMVs (pOMVs, circles) and biofilm derived OMVs (bOMVs, squares), represented as a percentage of the total OMV population. pOMVs are represented by circles (dark gray bars), and bOMVs are represented by squares (light gray bars). Data are presented as means ± SEM of *n* = 5 biological replicates. *, *P < *0.05; **, *P < *0.01; ***, *P < *0.001; ns, not significant (Student's *t* test). (F) The number of P. aeruginosa PAO9503 pOMVs (circles, dark gray bar) and bOMVs (squares, light gray bar) per 10^8^ bacteria in individual cultures at the time OMVs were collected was determined by NTA. Data are means ± SEM of *n* = 4 biological replicates. ns, not significant (Student's *t* test).

We first examined the morphology and size of biofilm and planktonic-derived P. aeruginosa OMVs using TEM and NTA. Planktonic and biofilm-derived P. aeruginosa OMVs were similar in morphology and were heterogeneous in size when examined by TEM (Fig. 3A and B). Examination of the size and concentration of particles within planktonic and biofilm-derived OMV samples using NTA revealed that both planktonic-derived OMVs (pOMVs) and biofilm-derived OMVs (bOMVs) ranged from approximately 50 to 400 nm in size, with the predominant OMV population ranging between 100 and 200 nm for both OMV types (Fig. 3C and D). Further comparison revealed that bOMVs were significantly smaller than pOMVs, with a larger proportion of bOMVs ranging between 10 and 100 nm in size, whereas a larger proportion of pOMVs were between 200 and 400 nm in size (Fig. 3E). Although bOMVs were smaller overall, an equivalent number of OMVs were isolated from both planktonic-grown and biofilm cultures of P. aeruginosa per 10^8^ bacteria, suggesting that altered bacterial growth conditions did not influence OMV quantity (Fig. 3F).

### Biofilm-derived P. aeruginosa OMVs contain more surface-associated DNA compared to planktonic-derived OMVs.

**FIG 4:**
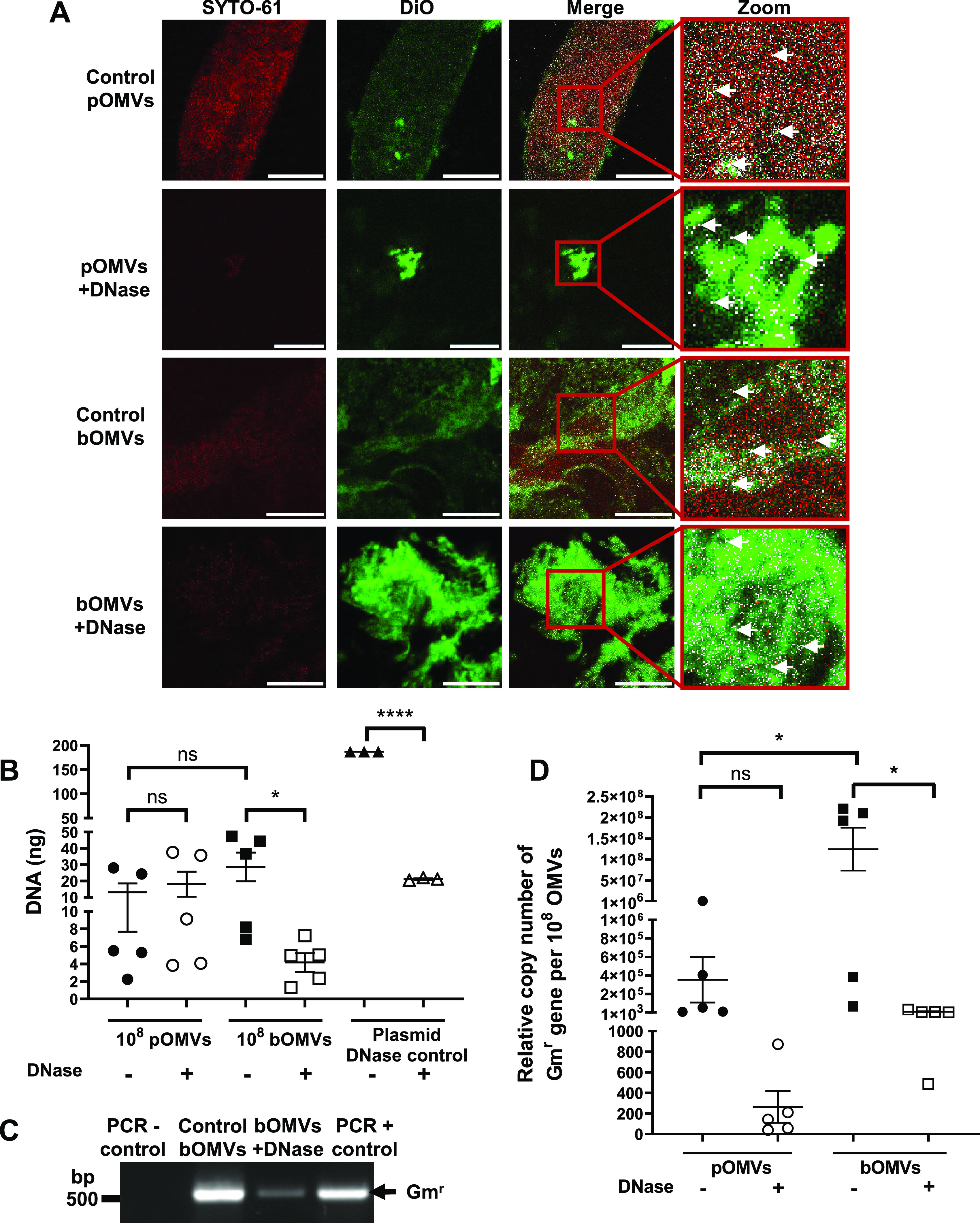
Biofilm-derived P. aeruginosa PAO9503 OMVs package plasmid DNA encoding antibiotic resistance. (A) Control and DNase-treated P. aeruginosa PAO9503 pBBR1MCS-5 planktonic-derived OMVs (pOMVs) and biofilm-derived OMVs (bOMVs) were stained with the lipophilic stain DiO (green) and the membrane-permeable DNA stain SYTO-61 (red) to observe DNA associated with OMVs and examined using confocal microscopy. Red boxes show the area that is magnified from the merged images (zoom). Areas of colocalization are shown in white and are indicated by white arrows. Images are representative of *n* = 3 biological replicates. Scale bar, 10 μm. (B) DNA associated with P. aeruginosa PAO9503 pBBR1MCS-5 control (−; filled shapes) and DNase-treated (+; open shapes) pOMVs (circles) and bOMVs (squares) was quantified using Qubit and is presented in nanograms of DNA per 10^8^ OMVs. pBBR1MCS-5 plasmid DNA was used as a DNase control (plasmid DNase control; triangles). Data shown are means ± SEM of *n* ≥ 3 biological replicates. ns, not significant; *, *P < *0.05; ****, *P < *0.0001 (Student’s t tests). (C) PCR of control (control bOMVs) or DNase-treated bOMVs (bOMVs + DNase) isolated from biofilm cultures of PAO9503 pBBR1MCS-5 using primers specific for the gentamicin resistance cassette (Gm^r^). H_2_O was used as a negative control (PCR − control) and pBBR1MCS-5 was used as a positive control (PCR + control). Arrow represents the gentamicin cassette amplicon (Gm^r^, 500 bp). Image is representative of *n* = 3 biological replicates. (D) The relative copy number of the Gm^r^ gene from pBBR1MCS-5, as a measure of plasmid copy number, was calculated per 10^8^ OMVs obtained from planktonic PAO9503 pBBR1MCS-5 (pOMVs; circles) or PAO9503 pBBR1MCS-5 biofilm cultures (bOMVs; squares). OMVs were left untreated (−; filled shapes) or DNase treated (+; open shapes) prior to plasmid quantification by qPCR. Data are means ± SEM of *n* = 5 biological replicates. ns, not significant; *, *P < *0.05 (one-way ANOVA with Tukey’s multiple comparisons).

Next, we examined the ability of pOMVs and bOMVs to package DNA, including plasmid DNA. To visualize the total DNA associated with pOMVs and bOMVs, DNase-treated and control OMVs were stained using SYTO-61, in addition to the lipophilic stain DiO to stain the OMV membrane. SYTO-61 staining of DNA was associated with both DiO-labeled control pOMVs and control bOMVs, indicating there was an association of DNA with OMVs produced by bacteria during conditions of planktonic and biofilm growth (Fig. 4A). However, reduced DNA staining was associated with both DNase-treated pOMVs and bOMVs compared to their controls, suggesting that DNA was predominantly located on the exterior surface of pOMVs and bOMVs (Fig. 4A).

To determine if DNase treatment significantly reduced the amount of DNA associated with the exterior surfaces of pOMVs and bOMVs, we quantified the amount of DNA associated with 10^8^ non-treated control or DNase-treated pOMVs and bOMVs. As a control, pBBR1MCS-5 plasmid DNA was treated with DNase to verify the efficiency of the DNase treatment. Quantification of the DNA cargo associated with OMVs revealed that there was no significant difference in the total amount of DNA associated with either pOMVs or bOMVs (Fig. 4B). Furthermore, DNase treatment did not significantly reduce the amount of total DNA associated with 10^8^ PAO9503 pOMVs (Fig. 4B), corroborating our findings obtained using control and DNase-treated planktonic-derived OMVs isolated from P. aeruginosa PAO9505 (Fig. 1C). In contrast, DNase treatment significantly reduced the amount of DNA associated with 10^8^ PAO9503 bOMVs compared to non-treated control PAO9503 bOMVs, revealing that a significant amount of DNA was associated with the exterior surface of bOMVs (Fig. 4B) (*P < *0.05).

Next, we used PCR to determine if there were any differences in the ability of pOMVs and bOMVs to package and protect plasmid DNA. PCR using primers specific for the Gm^r^ cassette contained within pBBR1MCS-5 revealed that plasmid DNA was associated with both control bOMVs and DNase-treated bOMVs, identifying that plasmid DNA was packaged and protected from DNase degradation within the lumen of bOMVs (Fig. 4C). Furthermore, the quantity of pBBR1MCS-5 associated with control and DNase-treated pOMVs and bOMVs was determined using qPCR (Fig. 4D). We found that 10^8^ PAO9503 bOMVs contained significantly more plasmid DNA than 10^8^ PAO9503 pOMVs (Fig. 4D) (*P < *0.05), as indicated via quantification of the Gm^r^ gene by qPCR, and that DNase treatment of bOMVs significantly reduced their plasmid copy number, suggesting that the majority of plasmid DNA associated with bOMVs was located on their exterior surface (Fig. 4D) (*P < *0.05). In comparison, DNase treatment reduced the relative amount of the Gm^r^ gene associated with PAO9503 pOMVs, suggesting the presence of plasmid DNA on the exterior surface of pOMVs; however, this reduction was not statistically significant. Collectively, these findings revealed that bOMVs are associated with more plasmid DNA than pOMVs and that DNA is mostly associated with the exterior surface of bOMVs, suggesting that bacterial growth conditions may affect the type and location of DNA packaged into OMVs.

### P. aeruginosa biofilm-derived OMVs are more efficient in the transformation of recipient bacteria than planktonic-derived OMVs.

**FIG 5:**
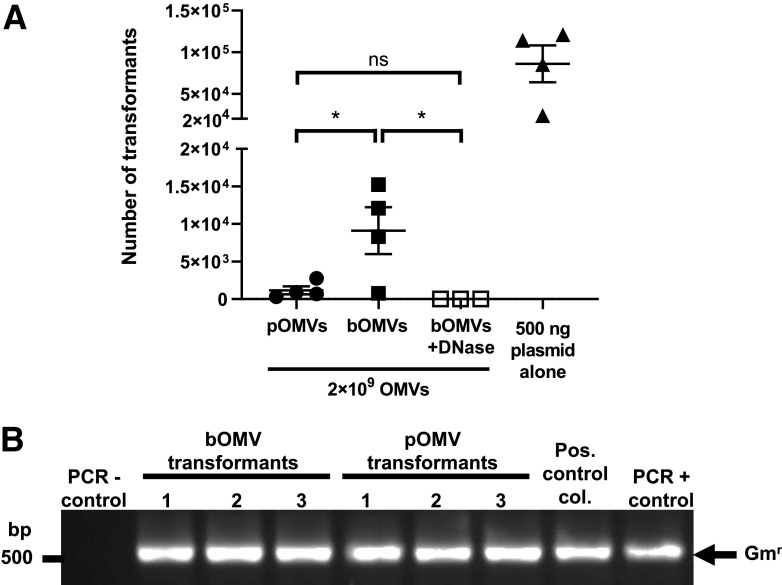
Biofilm-derived P. aeruginosa PAO9503 OMVs are more efficient at mediating horizontal gene transfer of plasmid DNA encoding antibiotic resistance than planktonic-derived P. aeruginosa PAO9503 OMVs. (A) The number of transformants resulting from transformation with either 2 × 10^9^ planktonic-derived PAO9503 pBBR1MCS-5 OMVs (pOMVs; closed circles), control non-treated PAO9503 pBBR1MCS-5 biofilm-derived OMVs (bOMVs; filled squares), DNase-treated PAO9503 pBBR1MCS-5 biofilm-derived OMVs (bOMVs + DNase; open squares), or 500 ng of plasmid DNA alone (filled triangles) are shown. Data shown are means ± SEM of *n* ≥ 3 biological replicates. ns, not significant; *, *P < *0.05 (one-way ANOVA with Tukey’s multiple comparisons). (B) PAO9505 transformants resulting from transformation using either non-treated P. aeruginosa PAO9503 biofilm-derived OMVs or PAO9503 planktonic-derived OMVs, both harboring pBBR1MCS-5, were confirmed by performing colony PCR using primers specific for the pBBR1MCS-5 gentamicin cassette. A positive plasmid control transformant colony (pos. control col.) and 1 ng pBBR1MCS-5 DNA (PCR + control) were used as positive controls. A non-transformed PAO9505 colony was used as a negative control for the PCR (PCR - control). Arrow indicates the gentamicin cassette amplicon (Gm^r^; 500 bp). Image is representative of *n* = 4 biological replicates.

To determine whether OMVs isolated from biofilm or planktonic growth conditions had different transformation efficiencies, control and DNase-treated OMVs isolated from both biofilm and planktonic cultures of PAO9503 pBBR1MCS-5 were added to competent P. aeruginosa PAO9505 bacteria, and the transformation efficiency was determined. For each transformation, 2 × 10^9^ OMVs were added to recipient bacteria, whereas the addition of 500 ng of pBBR1MCS-5 plasmid DNA or TE buffer to recipient PAO9505 bacteria served as positive or negative controls, respectively. Transformants were successfully obtained when P. aeruginosa PAO9505 was transformed using pBBR1MCS-5 plasmid DNA (Fig. 5A). In addition, transformation was observed with the addition of PAO9503 pOMVs harboring pBBR1MCS-5, resulting in an average of 1.16 × 10^3^ transformants (Fig. 5A). Transformation of recipient P. aeruginosa PAO9505 was also observed when bacteria were transformed with an equivalent number of bOMVs, with an average of 3.64 × 10^4^ transformants, which was significantly more efficient than transformation with pOMVs (Fig. 5A) (*P < *0.05). Only a single transformant was obtained as a result of transformation with DNase-treated bOMVs from a single experiment, and this result could not be replicated in two subsequent additional independent transformations using DNase-treated bOMVs, highlighting that this transformation event occurred at a very low frequency (Fig. 5A). Taken together, these findings revealed that although pOMVs and bOMVs contain similar amounts of total DNA, bOMVs are significantly more efficient in transforming recipient P. aeruginosa (Fig. 5A) (*P < *0.05). We confirmed the presence of pBBR1MCS-5 within all transformant colonies by extracting plasmid DNA from resulting transformants and amplifying the Gm^r^ cassette by PCR (Fig. 5B).

Overall, our findings showed that bOMVs are more efficient at transforming P. aeruginosa compared to pOMVs, highlighting that bacterial growth conditions can affect the packaging of DNA associated with OMVs and their ability to facilitate HGT. Although OMV-mediated transformation is a low-frequency event and the exact mechanisms are unclear, these findings revealed that OMV-mediated HGT is an effective mechanism for the transfer of plasmid-encoded antibiotic resistance genes between bacteria.

## DISCUSSION

BMVs, including OMVs produced by Gram-negative bacteria and MVs produced by Gram-positive bacteria, are known to package and protect DNA and can mediate HGT between bacteria (4–9, 11, 12, 18, 21). In addition to the ability of BMVs to package DNA, several studies have reported that BMVs package proteins, lipids, and toxins (22, 27, 29, 30), for which their packaging can be altered by bacterial growth conditions, such as growth during planktonic or biofilm conditions (22, 23, 27). Although OMV-associated DNA is thought to be integral for stabilization and maintenance of P. aeruginosa biofilm structures (28, 31), the potential for P. aeruginosa pOMVs and bOMVs to transfer DNA to recipient bacteria has not been previously demonstrated.

Several studies have shown that many species of bacteria grown as planktonic cultures release BMVs containing plasmid DNA that can mediate HGT. This includes OMVs produced by Neisseria gonorrhoeae that were shown to package plasmid DNA and were able to mediate HGT between different N. gonorrhoeae strains (4). Acinetobacter species have also been shown to release OMVs that package and protect plasmid DNA and can transfer their plasmid cargo to recipient Acinetobacter species (8, 9). Additionally, OMVs produced by pathogenic E. coli O157:H7 were shown to transfer plasmid DNA encoding green fluorescent protein to recipient E. coli JM109 and could also mediate interspecies HGT to Salmonella enterica serovar Enteritidis bacteria (6). OMVs produced by another pathogenic strain of E. coli, O104:H4, were found to package plasmid DNA encoding beta-lactamase that could be transferred to several enteric bacteria, including E. coli, Salmonella enterica serovar Typhimurium, Shigella sonnei, Shigella flexneri, Klebsiella pneumoniae, Enterobacter cloacae, and Proteus mirabilis, resulting in the spread of antibiotic resistance (12). In this way, BMVs have been shown to contribute to the distribution of plasmid DNA in several bacterial species, in both intra- and interspecies manners.

To date, limited studies have characterized the DNA cargo of P. aeruginosa OMVs (17, 21, 31), with one study having compared the association of DNA with P. aeruginosa pOMVs and bOMVs (31) and a second study examining the transformation abilities of P. aeruginosa pOMVs that contained plasmid DNA (21). Furthermore, a comparison of the ability of pOMVs and bOMVs produced by P. aeruginosa to mediate HGT has not been performed. Therefore, in this study we examined the DNA content of pOMVs and bOMVs produced by P. aeruginosa and determined their abilities to mediate HGT. Although BMVs have been shown to mediate HGT, the frequency whereby this process occurs is at a low rate and is more variable compared to conjugation, transformation, and transduction (32). Therefore, due to the difficulty in studying low-frequency BMV-mediated HGT, we used a model of chemically competent P. aeruginosa to identify the ability of pOMVs and bOMVs to transfer plasmid DNA to recipient bacteria. We demonstrated that pOMVs contained plasmid DNA and were able to transform recipient P. aeruginosa. Moreover, we found that pOMVs were significantly more efficient in the transfer of plasmid DNA to recipient bacteria, based on their plasmid copy number, compared to transformation with plasmid DNA alone. We hypothesized that this may be due to P. aeruginosa OMVs being enriched in DNA and can therefore efficiently bind to and deliver their DNA to recipient bacteria, compared to the lower efficiency of recipient bacteria in the uptake of free plasmid DNA. These transformations were conducted using control non-DNase-treated OMVs, and therefore we hypothesized that the plasmid DNA transferred to recipient bacteria was associated with both the surface and within OMVs. To investigate if plasmid DNA contained within the OMV lumen alone could be transferred to recipient bacteria, we attempted to transform P. aeruginosa using DNase-treated pOMVs. However, despite numerous attempts, we were unable to transform P. aeruginosa using DNase-treated OMVs. This may have been due to multiple reasons, which include (i) DNase-treated OMVs containing significantly less plasmid DNA than control OMVs and therefore resulting in a lower frequency of transformation compared to control OMVs that was below our detection limit, or (ii) the inability of DNA within OMVs to enter recipient bacteria, which may require bacterial competence genes to facilitate transformation of P. aeruginosa (9, 26). Furthermore, we hypothesized that the ability of DNA contained within OMVs to enter and be utilized by recipient bacteria may be more complex compared to the uptake of DNA associated with the exterior surface of OMVs and its subsequent replication within recipient bacteria, and this requires further investigation.

Before conducting transformations with bOMVs to compare their efficiency to that of pOMVs, we isolated P. aeruginosa bOMVs and compared their size and DNA cargo to pOMVs. P. aeruginosa bOMVs were significantly smaller in size than pOMVs, in concert with previous studies comparing the sizes of pOMVs and bOMVs (27, 28). We determined that bOMVs contained a similar amount of surface-associated DNA compared to pOMVs, as no significant difference was observed. However, previous studies have observed greater amounts of DNA associated with bOMVs produced by P. aeruginosa PAO1 and by Helicobacter pylori compared to their pOMV counterparts (23, 31). This may be due to strain- and species-dependent differences in OMV cargo, as we have previously reported that OMVs produced by different strains vary in the quantity of their DNA, RNA, and protein cargo (33). Although we did not observe a significant difference between the overall amount of DNA associated with PAO9503 pOMVs and bOMVs, we found that bOMVs were associated with significantly more plasmid DNA copies than pOMVs. Interestingly, E. coli OMVs produced by an *nlpl* mutant strain, in which peptidoglycan synthesis and breakdown were altered, have also been shown to package more plasmid copies than wild-type E. coli OMVs, despite their smaller size, as determined by NTA (34). This may have been due to cytoplasmic leakage of DNA into the periplasm (34), or by the increased production of outer-inner membrane vesicles (OIMVs), which have been shown to package cytoplasmic cargo and have increased packaging of DNA (20). The production of OIMVs has also been demonstrated for P. aeruginosa (20); however, the presence of OIMVs in bOMV populations has not yet been examined. Furthermore, the origin of replication of plasmids has also been shown to affect their packaging within OMVs, in which high-copy-number plasmids have increased packaging within OMVs compared to low-copy-number plasmids (35). The plasmid pBBR1MCS-5 is a low-copy-number plasmid (24); however, future studies may examine the ability of high-copy-number plasmids to increase their association with OMVs and examine their frequency of OMV-mediated transformation. Collectively, this demonstrates that several factors, such as genetic regulation, bacterial growth factors, and mechanisms of OMV biogenesis, may regulate the packaging of plasmid DNA within OMVs.

Upon transforming recipient P. aeruginosa with an equivalent number of pOMVs and bOMVs, we found that bOMVs were significantly more efficient in mediating HGT than pOMVs. P. aeruginosa was recently shown to become naturally competent and able to take up DNA when grown as a biofilm (26); therefore, we also hypothesized that there may be other factors, such as the presence of competence proteins within bOMVs, that may enhance their transformation efficiency. It has been proposed that the expression of competence genes by bacteria such as pathogenic E. coli and Acinetobacter baumannii may enhance the uptake of DNA from OMVs (9, 36), indicating that competence proteins may increase BMV-mediated HGT efficiency. Furthermore, P. aeruginosa grown as a biofilm has been demonstrated to have different surface lipopolysaccharide (LPS) profiles compared to P. aeruginosa grown during planktonic conditions (37). Therefore, OMVs produced by biofilm or planktonic cultures of P. aeruginosa may also have altered LPS profiles, which may affect the surface charge of P. aeruginosa OMVs and their ability to bind to recipient bacteria, to ultimately transfer their DNA to recipient cells (16). This suggests that bacterial growth conditions, such as biofilm or planktonic conditions, may not only alter the packaging of DNA within BMVs but may also be a factor that affects the ability of BMVs to interact with recipient bacteria to mediate HGT. Furthermore, the OMVs used in this study were not subjected to purification in order to resemble OMVs found within a physiological setting; therefore, it is possible that biofilm matrix components may be contained within the bOMV sample, which may also affect their ability to interact with bacteria. However, many of these key questions remain unanswered, and they are the focus of future research that aims to elucidate the mechanisms by which OMVs can mediate HGT.

The process of OMV-mediated HGT is thought to occur independent of specific genes required for natural competence, phage transduction, and the type IV secretion system (34), and therefore OMV-mediated HGT remains a unique mechanism for the dissemination of genetic material between bacteria. The findings generated as part of this study advance our knowledge of the production of DNA-containing OMVs and how their DNA content may be affected due to their generation during different growth conditions. Although we showed that P. aeruginosa OMVs were able to transform recipient P. aeruginosa by transferring plasmid-encoded antibiotic resistance during ideal transformation conditions, the ability of OMVs to transform P. aeruginosa grown as biofilms is unknown and remains the focus of future research endeavors. Similarly, other bacteria may also be efficiently transformed by BMVs when grown as biofilms rather than under planktonic laboratory conditions, and this remains to be elucidated. Future characterization of the composition of BMVs produced by different bacterial species during various growth conditions and their ability to mediate HGT will provide novel insights into the roles and functions of BMVs in the dissemination of antibiotic resistance genes and may provide opportunities to limit BMV-mediated HGT.

## MATERIALS AND METHODS

### Bacterial strains and plasmids.

Pseudomonas aeruginosa strains PAO9503 and PAO9505, which are sensitive to gentamicin, were routinely cultured on nutrient agar (NA; Oxoid, USA), or in nutrient broth (NB) containing 100 μg/mL streptomycin or 100 μg/mL rifampin, respectively, and incubated at 37°C as previously described (38). P. aeruginosa grown in NB was incubated with shaking at 200 rpm for 16 h. The plasmid used in this study was pBBR1MCS-5 (24), encoding a gentamicin resistance (Gm^r^) cassette, and strains containing pBBR1MCS-5 were routinely cultured on NA containing 30 μg/mL gentamicin.

### Isolation of plasmid DNA.

Large-scale plasmid DNA preparations from P. aeruginosa were performed using a Plasmid Maxi kit (Qiagen, Germany), and plasmid DNA was extracted from bacterial transformants using the Monarch plasmid miniprep kit (New England Biolabs, USA). Purified DNA was quantified using the Qubit dsDNA assay kit (Invitrogen, USA).

### Production of OMVs from planktonic cultures.

P. aeruginosa OMVs were produced and isolated using our established techniques (39). In brief, NB was inoculated using a 1:100 (vol/vol) dilution of an overnight P. aeruginosa culture and incubated at 37°C with shaking at 200 rpm for 16 h until bacteria had reached the stationary phase of growth. Bacteria were then pelleted by centrifugation at 4,000 × *g* for 1 h, and supernatants were filtered using a 0.22-μm polyethersulfone (PES) vacuum filter (Sartorius, Germany) and then concentrated 5-fold using a VivaFlow 200 tangential filter (10,000 molecular weight cutoff [MWCO], PES; Sartorius, Germany). OMVs in the concentrated supernatant were then pelleted by ultracentrifugation at 100,000 × *g* for 2 h at 4°C using a P28S rotor and Hitachi CP100NX ultracentrifuge (Hitachi, Japan). The resulting OMV pellets were stored at −80°C. All OMV preparations were confirmed to be free of bacteria by plating on NA and by examination using transmission electron microscopy.

### Production of P. aeruginosa OMVs from biofilm cultures.

P. aeruginosa was grown as a biofilm culture as previously described (28). Briefly, 1 mL of P. aeruginosa grown in NB for 16 h was used to inoculate the surface of an NA plate. Inoculated NA plates were allowed to dry and were incubated at 37°C for 24 h to allow for biofilm formation. OMVs were isolated from P. aeruginosa biofilms using established techniques (28). In brief, 4 mL of phosphate-buffered saline (PBS) was added to each plate and used to collect the biofilm by gentle pipetting. The resuspended biofilm was incubated at 37°C for 2 h and pipetted gently to resuspend bacteria. The bacteria were then pelleted by centrifugation at 4,000 × *g* for 30 min, and the resulting OMV-containing supernatant was sequentially filtered using a 0.45-μm PES syringe filter and then a 0.22-μm PES syringe filter (Sartorius, Germany). OMVs contained in the filtered supernatant were pelleted by centrifugation at 100,000 × *g* for 2 h at 4°C, and the resulting OMVs were confirmed to be free of bacteria by plating on NA and by examination using TEM. OMVs were stored at −80°C until required.

### Transmission electron microscopy of OMVs.

TEM was performed as previously described (22). Briefly, carbon-coated TEM grids were preincubated with poly-l-lysine, incubated with OMVs for 10 min, and washed twice with PBS for 10 min. Samples were then fixed using 1% glutaraldehyde–PBS for 5 min, washed with distilled water, and stained using 2% uranyl oxalate and methylcellulose uranyl acetate. Samples were imaged using a Jeol JEM-2100 TEM equipped with two Gatan digital cameras.

### Nanoparticle tracking analysis.

Nanoparticle tracking analysis (NTA) was performed using ZetaView (Particle Metrix, Germany) or NanoSight NS300 3.2 (Malvern Instruments, United Kingdom) instruments as described previously (22, 40). For NanoSight NTA (22), purified OMVs were diluted in Dulbecco’s PBS (DPBS; Gibco, USA), and DPBS was also used as a blank. NTA was performed in three 60-s reads, at camera level 13, screen gain 1, and detection threshold 3. The average of the three reads was calculated and plotted as particle size versus number of particles per milliliter. Three biological replicates were performed for each sample and were all plotted as the mean + standard error of the mean (SEM). NTA was also performed using a ZetaView Basic (Particle Metrix, Germany) (40). OMVs were diluted in DPBS to a final volume of 1 mL. Instrument calibration was conducted prior to analysis using 102-nm polystyrene beads (Thermo Fisher, USA). The default software setting for small extracellular vesicles was selected. Measurements were taken using a 405-nm 68 m laser and CMOS camera scanning at 11 cell positions and captured according to the manufacturer’s default settings. Data were analyzed using the ZetaView built-in software version 8.05.12 SP1 with the following parameters: maximum area, 1,000; minimum area, 5; maximum brightness, 255; minimum brightness, 30; minimum trace length, 15. Three or more biological replicates were performed for each sample, and results were plotted as the mean + SEM.

### DNase treatment and quantitation of OMV-associated DNA.

OMVs were treated with Turbo DNase (Invitrogen, USA) as per the manufacturer’s instructions for rigorous DNase treatment, or OMVs were left non-treated as a control. For DNase treatment, 0.1 volumes of DNase buffer and 2 U of Turbo DNase were added to OMVs, and the mixtures were incubated at 37°C for 30 min. Next, another 2 U of Turbo DNase was added, and OMVs were further incubated at 37°C for 30 min. DNase was inactivated by the addition of 0.2 volumes of DNase-inactivating reagent, and the reaction mixture was incubated for 5 min at room temperature, mixing occasionally. DNase-treated OMVs were washed using PBS by centrifugation at 100,000 × *g*, at 4°C for 2 h, and resuspended in 50 to 200 μL of PBS. Plasmid DNA or genomic bacterial DNA was used as a DNase control to confirm the degradation of DNA. DNA associated with non-treated control OMVs or DNase-treated OMVs was quantified using the Qubit dsDNA assay kit (Invitrogen, USA).

### Fluorescent staining of OMVs and confocal microscopy.

To stain OMV-associated DNA (17, 40), 5 nM SYTO-61 (Invitrogen, USA) was added to 5 × 10^8^ OMVs in 50 mM Tris buffer and incubated for 30 min at room temperature protected from light. OMVs were then washed three times in 50 mM Tris buffer by centrifugation at 4,000 × *g* at 4°C using a 10-kDa MWCO centrifugal filter (Merck, USA) and resuspended in 150 μL of 50 mM Tris buffer. OMVs were costained using 0.01 mM lipophilic stain DiO (40, 41) and washed three times by centrifugation at 4,000 × *g* at 4°C using a 10-kDa MWCO centrifugal filter. Examination of DNA associated with OMVs by confocal microscopy was performed as previously described (17, 40). In brief, OMVs were added to poly-l-lysine-coated slides and incubated protected from light for 1 h. The remaining liquid was removed, the coverslips were mounted to microscope slides using VectaShield (Vector Laboratories, USA), and samples were imaged using a Zeiss LSM 780 laser scanning confocal microscope. Images were analyzed using ImageJ software.

### Detection of DNA associated with OMVs by PCR.

DNA associated with OMVs was amplified by PCR. Briefly, 50-μL reaction mixtures composed of 0.8 μM forward and reverse primers (gentamicin forward, 5′-CGCAGCAGCAACGATGTTAC-3′; gentamicin reverse, 5′-CGGTACTTGGGTCGATATC-3′; Sigma-Aldrich, USA), 1 mM MgCl_2_, 200 μM deoxynucleoside triphosphate mixture, GoTaq Flexi buffer (Promega, USA), 1 U of *Taq* polymerase (Promega, USA), and 1 μL of sample (1 ng/μL of plasmid DNA, or a small amount of a bacterial colony), made up to 50 μL MilliQ H_2_O. PCR was performed using a T100 thermal cycler (Bio-Rad, USA). PCR to detect the presence of the gentamicin cassette was performed using 35 cycles. Each cycle consisted of denaturing at 95°C for 60 s, annealing at 55°C for 60 s, and extension at 72°C for 75 s.

### Quantification of OMV-associated plasmid DNA.

qPCR of pBBR1MCS-5 plasmid contained within OMVs was performed as previously described (11). Briefly, qPCRs consisted of 20 μM forward and reverse primers (forward primer, 5′-TTCGGAGACGTAGCCACCTA-3′; reverse primer, 5′-CAACAACCGCTTCTTGGTCG-3′), SensiFAST probe mix (Bioline, United Kingdom), and 2 μL of sample (control or DNase-treated OMVs), plasmid DNA as a positive control, or MilliQ H_2_O as a negative control, and volumes were made up to 20 μL with MilliQ H_2_O in a 96-well qPCR plate (Bio-Rad, USA). qPCR was performed using a CFX Connect real-time PCR system (Bio-Rad, USA) with cycle parameters consisting of denaturing at 95°C for 30 s and annealing at 55°C for 15 s. Gm^r^ gene copies as a measure of plasmid copy number were calculated as previously described (42), using the following equation: [(6.022 × 10^23^ copies per mole) × (DNA amount in grams)]/[(DNA length in base pairs) × (660 grams per mole per base pair)]. Threshold cycle values were plotted against the log of their initial template copy numbers using a standard curve generated with defined concentrations of purified plasmid ranging from 1 × 10^−7 ^to 1.0 ng.

### Bacterial transformation using OMVs.

The method for OMV-mediated bacterial transformation was adapted from a previous study (21). Briefly, recipient P. aeruginosa was grown in NB overnight at 42°C using static conditions, and bacteria were then pelleted by centrifugation at 4,000 × *g* at 4°C for 30 min. Bacteria were resuspended in 500 μL of 10 mM CaCl_2_, pelleted by centrifugation at 13,000 × *g* for 1 min, resuspended in 400 μL of 10 mM CaCl_2_, pelleted, and then resuspended in 300 μL of 75 mM CaCl_2._ After pelleting, the bacteria were resuspended in 200 μL of 75 mM CaCl_2_ and were used immediately for transformation assays. For transformation studies, 200 μL of CaCl_2_-treated P. aeruginosa was incubated with OMVs containing 500 ng of DNA or 500 ng of plasmid DNA as a positive control, or with either 50 μL Tris-EDTA buffer or no buffer as negative controls, on ice for 1 h. Each transformation reaction mixture was then incubated at 42°C for 1 h statically, followed by 37°C for 1 h statically, and then at 37°C for 1 h with shaking at 200 rpm. Each transformation mixture was then added to 10 mL superoptimal culture (SOC) broth and allowed to recover at 37°C, with shaking at 200 rpm, for a further 21 h. The bacterial cultures were then centrifuged at 4,000 × *g* for 1 h, the bacterial pellet was resuspended in 1 mL of SOC medium, and 50 μL of bacteria was used to perform serial dilutions to calculate the CFU per milliliter. The remaining transformation culture was grown on NA containing 30 μg/mL gentamicin to select for transformants. Plates were incubated overnight at 37°C, and the resulting transformants were enumerated. Transformants were selected and subjected to colony PCR and were cultured in NB containing 30 μg/mL gentamicin for plasmid miniprep DNA purification.

### Calculating transformation efficiency.

Transformation efficiencies were calculated as previously described (11). Briefly, the number of colonies resulting from transformation with DNA-containing OMVs or plasmid DNA were enumerated and calculated as CFU per nanogram of OMV-associated DNA or plasmid DNA. The number of plasmid copies associated with OMVs used in each transformation was determined using qPCR. Transformation efficiency was calculated as the number of transformants per 10^8^ plasmid copies, as calculated using qPCR, divided by the number of CaCl_2_-competent recipient bacteria that were used in each transformation. To compare the effectiveness of transformation using an equivalent amount of planktonic-derived and biofilm-derived OMVs, transformation efficiency was calculated as the number of transformants resulting from transformation with 2 × 10^9^ OMVs, as quantified using ZetaView NTA.

### Statistical analysis.

Data analysis was performed using the statistics software GraphPad PRISM v9.2.0. All data are presented as means ± SEM unless otherwise stated. Statistical analyses were performed using data from three or more biological replicates, using a one-way analysis of variance (ANOVA) with Tukey’s multiple-comparison test, Mann-Whitney U test, or Student’s t tests.
